# Reperfused human umbilical cords as an *ex vivo* model for ECMO cannulation in artificial placenta technology

**DOI:** 10.3389/fbioe.2025.1663442

**Published:** 2025-10-21

**Authors:** Camila Hoyos Banchon, Jan Heyer, Juliette S. van Haren, Thorsten Orlikowsky, Ulrich Steinseifer, Sebastian V. Jansen, Mark Schoberer

**Affiliations:** ^1^ Pediatric Clinic, Neonatology Section, Faculty of Medicine, RWTH Aachen University and University Hospital, Aachen, Germany; ^2^ Department of Cardiovascular Engineering, Institute of Applied Medical Engineering, Helmholtz Institute, RWTH Aachen University and University Hospital, Faculty of Medicine, Aachen, Germany; ^3^ Department of Industrial Design, Eindhoven University of Technology, Eindhoven, Netherlands

**Keywords:** extremely preterm infants, artificial placenta, *ex vivo* umbilical cord setup, ECMO cannulation, human umbilical cord

## Abstract

**Background:**

Establishing an extracorporeal circuit for artificial placenta technologies requires the cannulation of the umbilical vessels with large-bore cannulas. So far, this procedure could only be performed in large-animal models. We present the methodology and a novel test setup for reperfusion of human umbilical cords to develop and train cannulation procedures.

**Methods:**

67 umbilical cords and placentas were harvested during cesarian sections, 15 cm umbilical cord specimen and fetal blood were used for these experiments. All three umbilical vessels were flushed, terminal cannulated, and connected to a circuit. We performed systematic validation tests on reperfusion of 15 umbilical cord specimens, followed by an ECMO cannulation of all three reperfused vessels.

**Results:**

The terminal cannulation succeeded in 43 of 67 cases. 15 of these were included in the validation study. A physiological flow of 71.2 mL/min ±3.9 mL/min (mean ± SD) could be established. ECMO Cannulation of at least one artery and one vein succeeded in 9 of 15 cases, and cannulation of all three vessels in five cases.

**Conclusion:**

Reperfusion of human umbilical cord specimens with fetal blood can provide a biosimilar model for the development, testing, and training of umbilical vessel cannulation with large-bore cannulas.

## Graphical Abstract

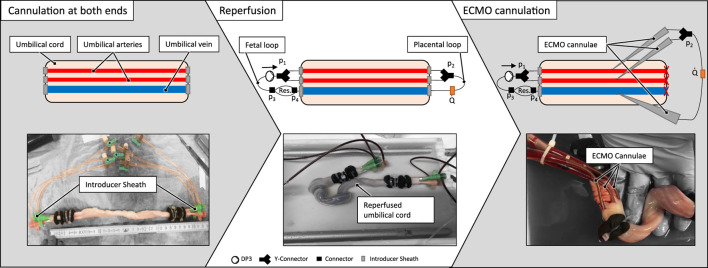

## Introduction

About 13 million preterm neonates are born worldwide every year (Ohuma et al., 2023). One million of these infants die from the pathology which enforces preterm birth and from complications of prematurity itself. The most immature patient group are extremely preterm infants (EPI) with a gestational age of less than 28 weeks. EPI regularly need neonatal intensive care treatment to allow survival despite their extreme immaturity (Blencowe et al., 2013; Dougherty et al., 2022). Recent progress in clinical treatment, especially non-invasive respiratory support, has significantly improved outcomes in this patient group (Göpel et al., 2015). Nonetheless, pulmonary gas exchange, including mechanical ventilation, continues to cause lifelong organ damage, particularly to the immature lung. The most important in this context is Bronchopulmonary Dysplasia (BPD), which affects more than half of the extremely preterm infants (Hurskainen et al., 2021; Regin et al., 2022).

Artificial amnion and placenta technologies (AAPT) are promising approaches in reducing the prematurity-related lung injury (van der Hout-van der Jagt et al., 2021). In lamb and piglet models, several working groups have successfully achieved the transfer of extremely immature fetuses to extrauterine, fluid-based environments (Hornick et al., 2016; Miura et al., 2016; Ch et al., 2021; Eixarch et al., 2023; Charest-Pekeski et al., 2022; Hornick et al., 2018). Artificial placentas are also developed as rescue treatment for preterm respiratory failure in conventional neonatal intensive care treatment and for use in an air based environment with occlusion of the airway (Omecinski et al., 2023; Kosaka et al., 2025; Sanchez-Martinez et al., 2023; van et al., 2025; Church et al., 2018; Schoberer et al., 2012).

Gas exchange during AAPT happens extracorporeally within membrane oxygenators (ECMO) which are connected to the fetus via the umbilical cord. Large bore cannulation of umbilical vessels is an essential technique for this approach. Cannulation of umbilical vessels for AAPT treatment will require the development of cannulas specifically designed for this purpose. These need to fit tightly into the vessel, resist traction and have a low resistance. Reliable long-term operability is crucial in this treatment because failure is immediately life-threatening and can cause lifelong neurocognitive impairment. Although the hypoxia tolerance of the newborn and especially the preterm brain is probably longer than the 3–5 min in normothermic adults (Singer, 1999), we assume that the cannulation time should not exceed 3 minutes to be regarded as safe for ethical considerations. Therefore, the EXIT umbilical vessel cannulation procedure ought to be developed, time optimized and trained in animal models or in *ex vivo* human test setups before being introduced into clinical practice. The human *ex vivo* model, in this case, is superior to the animal models due to animal ethics and biosimilarity considerations. The umbilical cords of piglets and lamb differ from human ones in anatomy regarding number of vessels, vascular diameter, and tissue consistency (Luis Manuel, 2018; Randall, 1989; Jordan, 1919).

Clinical and technical experience with ECMO-cannulation of umbilical vessels is scarce. To date, there is a lack of short and long-term data on human umbilical cord use for extracorporeal circulation. Cornish Et Al. (1991) performed cannulation of human umbilical veins as a supplemental drainage to the jugular venous access during ECMO rescue therapy on three subjects (CORNISH et al., 1991). Bjøro and Stray-Pedersen, (1986) introduced the concept of utilizing reperfused human umbilical cord cutoffs to draw conclusions about physiological *in vivo* conditions for medication purposes (Bjøro and Stray-Pedersen, 1986). The effect of vasoactive substances on the umbilical vascular tone was tested, confirming the possibility of reviving umbilical tissue (Bjøro and Stray-Pedersen, 1986). The cutoffs were reperfused in a test stand using a low-viscosity perfusate with a pH value similar to amniotic fluid (Bjøro and Stray-Pedersen, 1986). This test stand was, however, not intended for cannulation testing nor the simulation of extracorporeal life support (ECLS) (Bjøro and Stray-Pedersen, 1986). Only unphysiological low flow rates could be achieved using this test setup (Bjøro and Stray-Pedersen, 1986).

We present an *ex vivo* human umbilical cord test setup using autologous placental blood intended for the simulation and testing of human umbilical ECMO cannulation. The *ex vivo* model of blood-perfused human-umbilical cords features adjustable blood flow and–pressure. We were able to show the successful parallel cannulation of all three human umbilical vessels with commercially available ECMO cannulae. To our knowledge, for the first time.

## Methods

### Ethics, subjects and samples

Donated human umbilical cords were collected from the centralized biomaterial bank (cBMB) of RWTH Aachen University directly upon delivery via caesarean section. Written maternal consent was obtained in all cases. Exclusion criteria were defined as minor legal age, refusal of consent, and maternal or fetal infectious diseases. This procedure was specifically approved for this project (CORDLUA) by the ethics committee of the medical faculty of RWTH Aachen University (EK 206/09). Data from the mother, the neonate, and the cord were pseudonymized using a sample code from the cBMB. The lookup table is only accessible to the project physicians and the cBMB.

The harvested umbilical cord was clamped intraoperatively as close to the neonate as possible, severed and subsequently clamped again, leaving a short segment for clinical pH measurement. The remaining, longer portion of the cord on the placental side was used for this study. The placenta with the attached umbilical cord was immediately transferred to a workbench after the cesarean section. At the workbench, the clamped umbilical cord was severed from the placenta. Fetal blood from the placenta and the umbilical cord was collected into a tube and was immediately heparinized with 15.000 IU/L of heparin sodium (Heparin-Natrium-5000-ratiopharm Ratiopharm, Ulm, Germany). The cord segment was then rinsed superficially and manually extruded to remove the remaining blood and possible clots. The three umbilical vessels were probed and flushed with 2.5% papaverine solution (PAVERON^®^ N 25 mg/mL, Linden Arzneimittel-Vertrieb-GmbH, Heuchelheim, Germany). A blunt pediatric endotracheal tube replacement obturator (G05170: Cook Mizus Mettro Endotracheal Tube Replacement Obturator 7F X 70cm for 5 mm + ID, Cook Medical, Bloomington, Indiana, USA) was inserted for progressive probing up to 1 cm, and a 0.7 mm (22G) flexible catheter (Leaderflex Vascular catheter, Vygon, Ecouen, France) was used for flushing. Excluded were cord samples with a length of less than 15 cm, with umbilical vessels, which could either not be cannulated with cannulas larger than 15G or which were not free of clots after extrusion and flushing. Moreover, cases in which the harvested placental blood volume was less than 20 mL were excluded.

Included umbilical cords were prepared for the experiments through terminal cannulation on both ends in all vessels, at least in one artery and one vein. Systematic training was conducted in order to practice the harvesting procedure as well as the terminal cannulation. Cannulae of increasing sizes were introduced into each vessel of the cord cut off, starting with 15 G central venous catheters until 6 Fr sheaths could be introduced into the arteries and 7 Fr sheaths into the vein. The vein, being the largest and least constricted vessel, was cannulated first. The following commercially available introducer sheaths were used for the terminal cannulation: Terumo 7Fr, Terumo 6Fr, Terumo 5Fr, Terumo 4Fr (Terumo Radifocus™ Introducer II Pediatric Kit B Introducer Sheath, 6Fr (RS + B60K10MQ) arteries, 7Fr (0.035 (0.89 mm), RS + B70K10MQ) vein, Terumo Corporation, Tokyo, Japan). When the introducer sheaths were too large, 14 G single-lumen central venous catheters (Arrow ES-04700, Teleflex Medical, Athlone, Ireland) were used. The sheaths as well as the CVCs had previously been shortened to 6.5 cm.

The terminally inserted sheaths were fixated and sealed for tightness and to avoid dislocation through traction or pressure. This was achieved by wrapping the cannulated ends of the umbilical cord with self-adhesive acrylic compression tape (15 mm wide, 4–20 mm expansion, Soudal N.V., Leverkusen, Deutschland) and tightening the compression tape with cable ties (3 or 4 mm width). The tightening force was standardized using cable tie pliers (GS2B, Panduit Illinois, USA). A load of 12 ± 3 lbs was applied to the cable ties.

Successful terminal cannulation was verified through repeated flushing with papaverine solution, simultaneously ensuring a pharmacological counteraction of the vasospasm. Patency was assumed when the vessel could be flushed with low resistance and visible fluid outflow.

### Test setup

**FIGURE 1:**
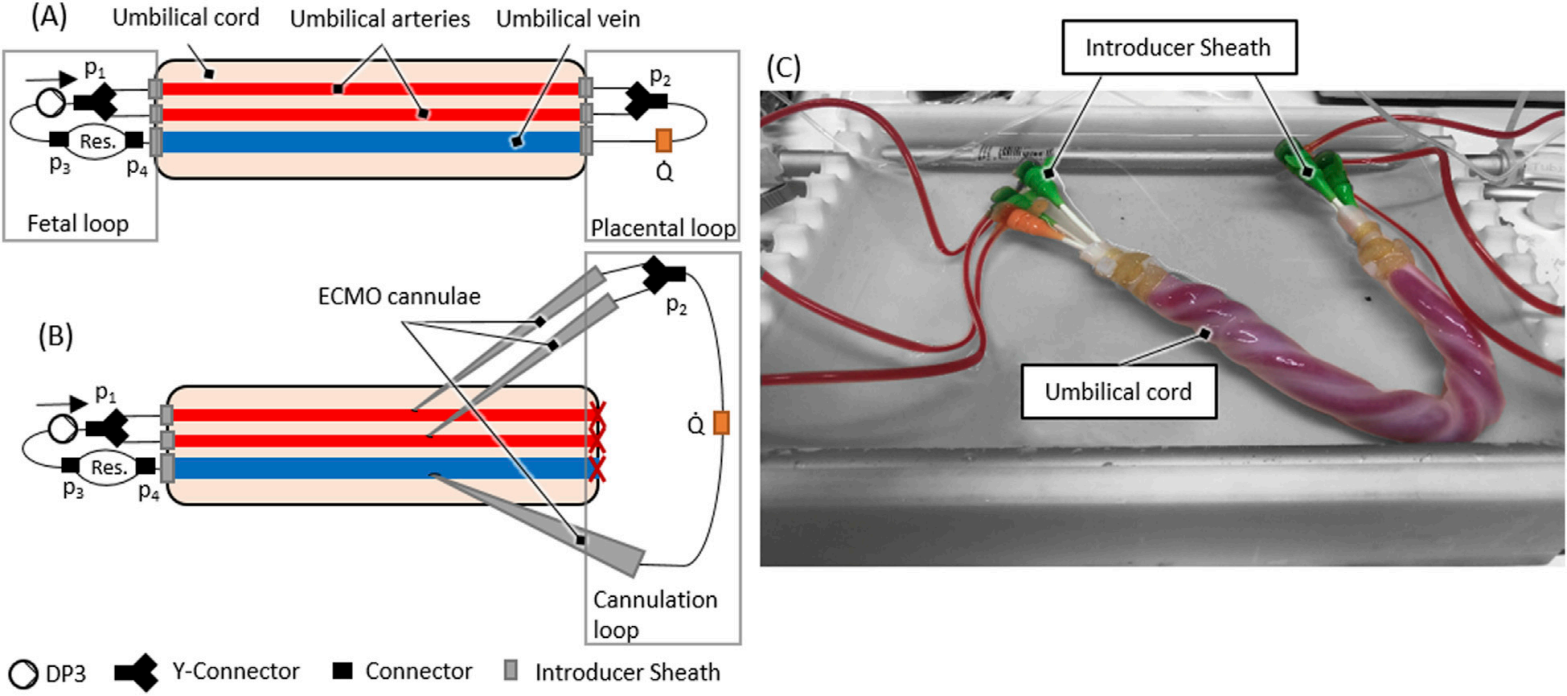
**(A)** Scheme of the *ex vivo* test setup **(B)** Scheme of an ECMO cannulated umbilical cord within the *ex vivo* test setup **(C)** Reperfused umbilical cord within the *ex vivo* setup as schematically presented in **(A)**.

The test setup incorporated an artificial blood-circuit and a thermostat-controlled warming bath for immersion of the umbilical cord specimen. The artificial blood circuit mimicking the native fetoplacental blood circuit consisted of two flow loops (‘fetal’ loop and ‘placental’ loop) and the umbilical vessels in between these loops. The setup is shown in Figure 1. The fetal blood loop incorporates a centrifugal pump (Delta Stream DP3, Medos Medizintechnik, Stolberg, Germany), mimicking the function of the fetal heart and consisting further of a blood reservoir to compensate for volume changes by the extension of the umbilical vessels during reperfusion. The placental flow loop consisted of a bypass from the arteries to the vein and the flow measurement (Transonic, New York, USA) of the circuit (Figure 1). Pressures were measured at four points within the circuit (p_1_, p_2_, p_3_ and p_4_, cf. Figure 1). Measurement points p_1_ and p_2_ were in flow direction, before and behind the branchings of the arterial route. Pressure p_1_ represents the blood pressure generated by the pump. The delta between p_1_ and p_2_ is the pressure loss caused by the resistance of the artery/-ies, the (two to four) arterial sheath-catheters, stop-cocks, tubing and connectors. Measurement points p_3_ and p_4_ were before and behind the blood reservoir. The delta between p_2_ and p_3_ shows the pressure loss caused by the resistance of the vein, the two venous sheath catheters, stop-cocks, tubing and connectors. The delta between p_3_ and p_4_ measures the pressure loss over the reservoir. The delta between p_1_ and p_4_ shows the total pressure loss over the complete system.

Both flow loops were primed separately with the harvested placental blood. If the harvested blood volume was lower than the minimum volume needed for the circuit (±56 mL), the placental blood was diluted using a sodium chloride solution (0.9% isotonic saline solution, B. Braun, Melsungen, Germany) until the necessary volume was reached. The terminal cannulation was connected to the blood-primed fetal- and placental loop. Subsequently, the circuit, including the umbilical cord sample, was immersed into a fluid bath filled with heated (37 °C) ringer solution (Ringer-Infusionslösung B. Braun, Melsungen, Germany).

### Validation study

The validation of the presented *ex vivo* test setup had two main objectives: to demonstrate that it is possible to reperfuse a human umbilical cord specimen at flow rates which are physiological for a fetus of 24 weeks of gestational age (GA), and to demonstrate that it is possible to introduce commercially available, ECMO cannulae into the umbilical vessels during reperfusion.

According to (Kiserud and Acharya, 2004; Kiserud et al., 2006), the umbilical vascular flow of a fetus at 24 weeks of GA ranges from 60 to 80 mL/min (Kiserud and Acharya, 2004; Kiserud et al., 2006). Hence, the target flow rate interval for our study was set to 70 ± 10 mL/min.

The blood flow, starting at 10 mL/min was progressively increased in steps of 10 mL/min every 5 min until the target flow was reached. This allowed the perfusion pressure to augment progressively and the vasospasm to dissolve fully. If a non-linear pressure change of sudden movement of the umbilical cord during the flow increase was observed, further flow increase was paused for 10 min. Reaching and keeping the target flow rate of 70 ± 10 mL/min with constant pressure values for a time period of at least 15 min was defined as the primary goal. The resulting pressure and blood flow data were analyzed by means of regression in R studio (Posit PBC, Boston, USA).

Subsequently, ECMO cannulation on the placental side of the reperfused umbilical cord was performed by insertion of large-bore ECMO-cannulae into each reperfused umbilical vessel. The vein was cannulated first, followed by the two arteries. The umbilical cords were incised progressively layer by layer using a no. 15 surgical scalpel. The preparation technique aimed to achieve the smallest possible incision to minimize blood loss as well as trauma to the vessel. The introducer was placed in the vessel lumen as soon as the vascular intima was opened, and the vessels were manually compressed. The introducer was used to ensure the correct depth of the incision and to “probe” the vessel. Subsequently, the introducer was removed, and the primed and connected ECMO cannula (DLP^®^ Pediatric One-Piece Arterial Cannula, 8Fr, Medtronic, Dublin, Ireland) was introduced into the vessel. Finally, the cannula was fixated identically to the technique described for the terminal cannulation.

These cannulae were then connected to a secondary cannulation loop representing the extracorporeal circuit in AAPT, and the placental flow loop connected to the initial terminal cannulation was shut off (cf. Figure 1B). The flow for the ECMO cannulation was then decreased from 70 mL/min to 30 mL/min to put a physiological placental pressure load of 30 mmHg (Wang and Zhao, 2010) on the cannulation. The flow rate of 30 mL/min to achieve this pressure loss (or a higher pressure loss) was determined in preliminary experiments. After successful cannulation, flow rate was again increased to 70 ± 10 mL/min to check for tightness of the ECMO cannulation.

## Results

A total number of 67 umbilical cords were harvested. 20 (29,8%) of the donated umbilical cords were excluded since they did not meet the length criteria of >15 cm. Two cords (3.0%) were excluded due to maternal infections, and an additional two (3.0%) due to handling mistakes in the cannulation process. 43 cords were cannulated terminally of which 28 cords were used for preliminary training purposes. This resulted in n = 15 umbilical cords being included in the validation study.

### Validation study

From the 15 umbilical cords included in this study, five umbilical cords could only be cannulated at one artery, and the vein (VA) and ten umbilical cords were successfully cannulated at both arteries and the vein (VAA). The used cannulae within each case are presented in Supplementary Material 1. On average a flow rate of 71.2 mL/min ±3.9 mL/min (mean ± SD) with constant pressures over 15 min could be successfully reached in all 15 experiments. In one experiment (No. 11), the pressure measurement failed, so no data on the pressure loss could be measured (see Table 1).

**TABLE 1:** Overview of child-related umbilical cord data and *ex vivo* setup pressure loss as well as vessels cannulated.

No.	Child weight	GA	Gender	Pressure loss in mmHg			Vessels cannulated
				Δp_V_	Δp_A_	Δp_Total_	
1	3,485	39.71	F	53	92	145	VA
2	3,720	38.29	M	54	91	145	VA
3	3,140	39.14	F	106	105	211	VA
4	3,710	39.29	M	58	58	116	VA
5	3,710	38.43	F	53	55	108	VA
6	2,530	37.14	M	94	40	134	VAA
7	3,279	38.00	F	84	23	107	VAA
8	3,270	39.27	M	35	46	81	VAA
9	3,060	38.71	M	73	51	124	VAA
10	3,360	39.00	M	42	20	62	VAA
11	2,970	37.00	F	--	--		VAA
12	2,605	38.57	F	47	24	71	VAA
13	3,080	39.57	M	32	20	52	VAA
14	4,400	38.86	M	90	48	138	VAA
15	3,830	38.83	M	97	40	137	VAA

**FIGURE 2:**
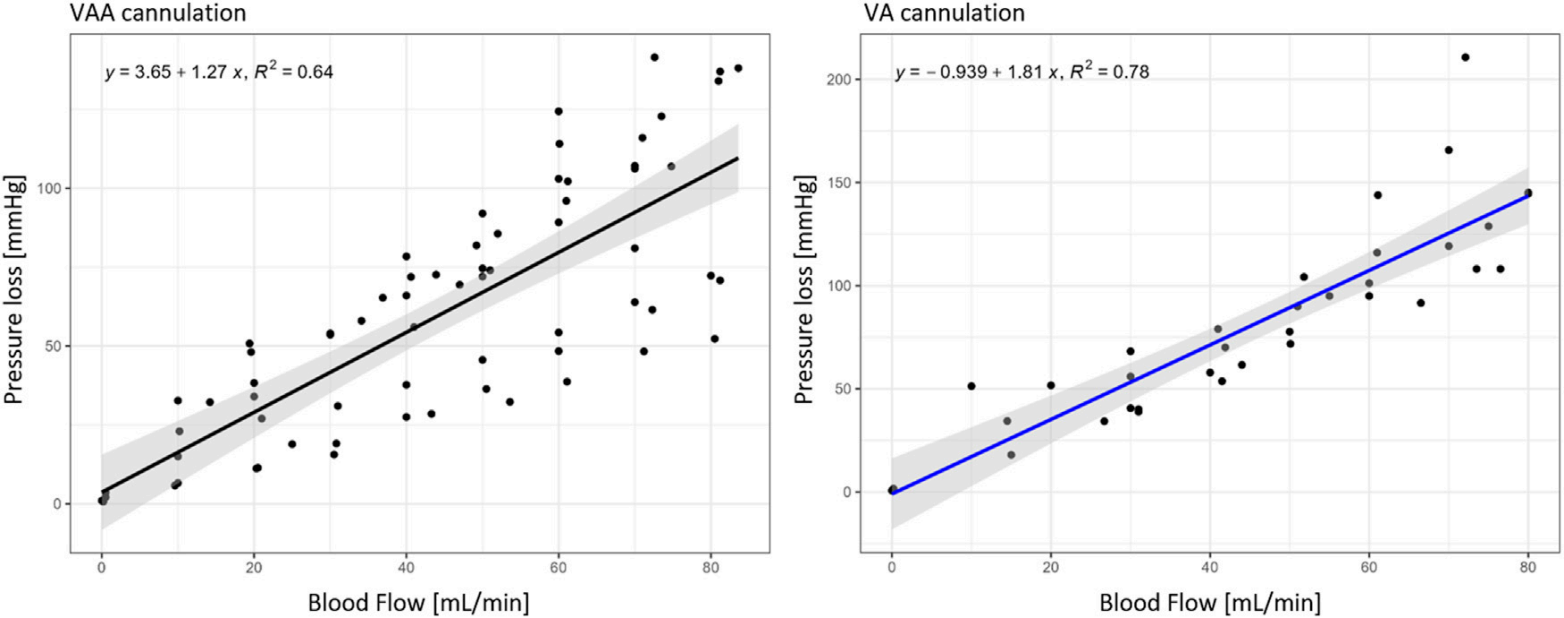
Pressure loss of VAA (n = 9) and VA cannulation (n = 5) within the target flow experiments in relation to the corresponding blood flows; Black regression line for VAA and blue for VA cannulation with the 95% interval in grey.

Duration until the establishment of the target flow rate was 36.6 min ±9.0 min (mean ± SD), the detailed perfusion times are also shown in Supplementary Material 1. The mean pressure loss was 51 ± 27 mmHg (mean ± SD) in the arteries and 66 mmHg ±24 mmHg (mean ± SD) over the vein. Pressure losses are shown in Table 1 for each individual experiment, together with corresponding subject characteristics of the newborn from which the cord was harvested (child weight, gestational age, gender and cannulated vessels). The total pressure loss of the circuit, defined as the pressure loss over the arteries and the vein, is plotted with the corresponding blood flows in Figure 2. A regression line with 95% percentiles is included in the plots.

**FIGURE 3:**
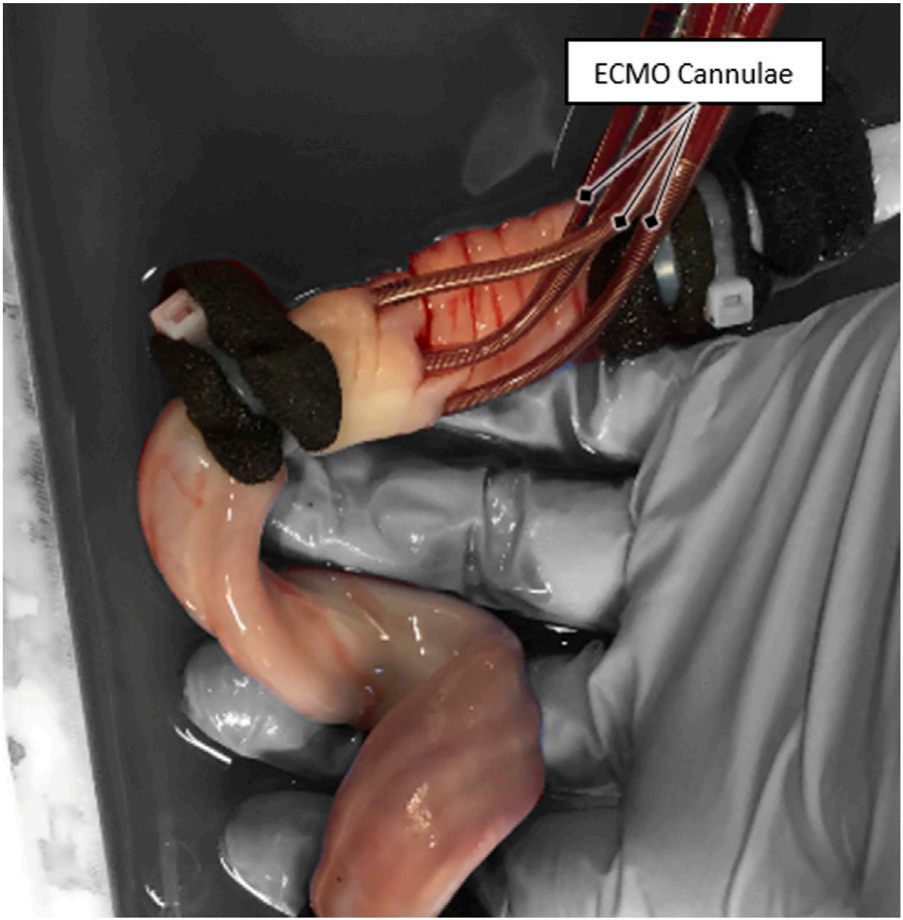
ECMO cannulation of the umbilical cord.

ECMO-cannulation was attempted on all 15 umbilical cords directly after the primary goal of reperfusion was reached. ECMO cannulation of both arteries and one vein was successful in five cases (33%), cannulation of one vein and one artery in another five cases (33%), and cannulation failed in the remaining five cases (33%). From the ten successfully cannulated cases, a blood-flow rate of 70 ± 10 mL/min over the newly established secondary cannulation loop was achieved in nine cases (90%). The mean total pressure loss at this flow rate was 49.6 mmHg ±2.8 mmHg (mean ± SD). A picture of a successful ECMO cannulation of an umbilical cord is shown in Figure 3.

In the five cases that were fully ECMO cannulated (VAA) in all three vessels, the maximum recorded cannulation time was 9.4 min, and the minimum cannulation time was 2.5 min.

In a supplementary single pilot experiment, a double ECMO cannulation of all three umbilical vessels can be reported using the test setup. With blood circulation through only the six large-bore ECMO cannulae, the total pressure loss was measured to be 9 mmHg at a flow rate of 85 mL/min.

## Discussion

This work describes the development and establishment of an *ex vivo* test setup which allows to perform cannulation experiments on reperfused human umbilical cord vessels. The method is novel and is suitable to close relevant gaps in translational research of artificial placenta technology. The presented setup and cannulation method enable a safe and ethically acceptable method for training in umbilical cord cannulation, particularly for AAPT or neonatal ECMO, with the umbilical cord serving as the access point. Compared to the experiments of Bjoro et al. (Bjøro and Stray-Pedersen, 1986), we developed a comprehensive circuit that includes arterial and venous umbilical vessels and allows for the addition of further circuit components, such as oxygenators. Pressure losses are still in the supraphysiological range but much closer to physiological pressures compared to the previously reported setup (Bjøro and Stray-Pedersen, 1986). Further improvements in terminal cannulation strategy (use of larger cannulae) might allow physiological pressures and flow in future studies. All material needed for these experiments can be harvested after childbirth from repelled tissue, so the method inherently carries low risk, is more ethical as it does not involve patients. It allows for the reduction of the need for animal studies. Moreover, it is superior to the quality of animal experiments for biosimilarity reasons: the anatomy of animal umbilical cords differs significantly from the human one, concerning the number and architecture of the vessels, their elasticity, vascularization, length, and tear resistance (Luis Manuel, 2018; Randall, 1989; Jordan, 1919).

Previous studies on umbilical cord cannulation by different research groups reported the general feasibility of cannulation procedures within their respective animal models. (Hornick et al., 2016; Charest-Pekeski et al., 2022; Hornick et al., 2018; Schoberer et al., 2012; Arens et al., 2011). With these results, the feasibility in humans was assumed. Hornick et al. suggested to evaluate the possible cannula diameter with sonographic measurements (Hornick et al., 2016). However, the translation of umbilical cannulation results from the animal model to the human neonate has been controversially discussed (Bie et al., 2021). Vasospasm and differences in vessel properties are reported as important challenges (Ch et al., 2021; Bie et al., 2021). With the *ex vivo* test setup presented in this work, the feasibility of ECMO cannulation in an *ex vivo* human umbilical cord was shown. The vasospasm of the human umbilical cord and its prevention may be examined in a reproducible laboratory environment under the given limitations by the *ex vivo* test stand. In addition, it is now possible to translate the results from animal models to a preclinical human setup in order to examine their validity.

Beyond artificial placenta technology (AAPT), the test setup could likewise be used to train and develop treatments and surgical techniques in fetal, perinatal and neonatal care, such as for scenarios like cordocentesis. To adequately train healthcare professionals with cannulation techniques in a high-fidelity setting, the environment can be adapted to such tasks by integrating the test setup into a maternal manikin with an artificial uterus. This way, human patient care practices can be trained, embedding cannulation within broader perinatal procedures rather than isolating it as a standalone skill.

While the medical simulation field already offers artificial umbilical cords (Sawyer et al., 2018), they are intended for neonatal care training. Also, integration of natural umbilical cords within artificial simulation setups has been described (Ghazali et al., 2023). However, in both cases, none exists for training umbilical cannulation for blood circuit establishment with AAPT.

In the process of establishing this novel approach, we had to use different commercially available vascular catheter devices for the different purposes. For the terminal cannulation, a cannula size of 7 Fr (arteries) and 8 Fr (vein) shortened to a length of 6.5 cm was found to be a viable compromise regarding cannulation time, insertion length, and the goal of atraumatic cannulation. Umbilical cord cannulation had to be trained in the sense of a progressive “learning curve”. Since a continuous improvement in outcome, time, and possible cannula sizes was observed during the training on the 28 umbilical cords, the cannulation skill improved. Further improvement could be achieved by, for instance, adding ultrasound guidance during the cannulation or by developing new cannulas specifically designed for umbilical cords. The technique is representative of a clinical skill that can be learned and trained. The total number of 43 terminally cannulated cords suggests the independence of terminal cannulation from umbilical cord anatomy, thereby opening broad possibilities for ethical clinical training and testing on human umbilical cords in a wide span of gestational ages.

AAPT applications aim to be a treatment alternative for extremely preterm neonates. In this study, most umbilical cords were obtained from term-born neonates with a gestational age (GA) above 36+0 weeks. At this GA, the mean vascular diameters are approximately 4.2 mm for the arteries and 8.2 mm for the vein, compared to 3.25 mm and 6.5 mm, respectively, at 24 weeks of GA (Kiserud and Acharya, 2004; Kiserud et al., 2006; Di Naro et al., 2001) Owing to the linear viscoelastic properties of the umbilical cord (Arens et al., 2011), cords from term neonates can still be perfused under the physiological flow and pressure conditions of a younger GA, thereby functionally mimicking earlier developmental stages. This approach avoids the need to harvest cords from preterm neonates and mitigates the associated ethical concerns. The observed flow rates and resulting vessel diameters in our experiments support the validity of this assumption. Since there is no change in the mechanical behavior of the umbilical cord, the same assumption can be made for umbilical cords with abnormalities, such as a single artery. Nevertheless, due to the exclusion of such umbilical cords from the proof-of-concept study, this cannot be verified.

The target flow could be established in all 15 included samples, showing the reproducibility of the setup. Corresponding pressure loss is, however, still supraphysiological and needs to be addressed in a future improved terminal cannulation strategy. VAA and VA pressure loss over blood flow can be described using linear regression (cf. Figure 2) to define operating conditions in future experiments.

ECMO cannulation of at least one artery and one vein was shown in ten out of 15 cases (66%). The fastest cannulation time was measured to be 2.45 min for all three vessels, showing that the required maximum duration of 3 min for cannulation is feasible. However, the sealing and fixation technique of the cannulae using cable ties and compression tape was aimed at a fast and tight connection for this feasibility study and was developed only for the laboratory use of the *ex vivo* test setup. Clinical application of ECMO cannulae to the umbilical cord and respective strategies against dislocation of the cannulas still need to be developed. The fixation was applied before the vessels were fully dilated, which could create a bending effect and cause higher resistance. The fixation method using a cable tie might have further caused trauma to both the cord and vessel wall. Histopathological examination of the reperfused vessels might clarify the options for optimization of the employed method.

The ECMO cannulation flow of 30 mL/min was chosen based on preliminary experiments to have a pressure load of at least 30 mmHg on the cannulae. The choice is verified by the pressure loss results illustrated in Figure 2. From the linear regression follows a pressure loss of 41.75 mmHg at 30 mL/min, which is slightly higher than the targeted 30 mmHg. Bypassing the *ex vivo* setup with up to six ECMO cannulae can reduce the pressure loss to 9 mmHg, making physiological operation points possible in this setup. The umbilical cord is perfused continuously instead of in a pulsatile manner as it is *in utero*. Preliminary tests using a pulsatile pump resulted in several suction events during flow rate ramp-up. The addition of pulsatility might be possible after a sufficient dilation of the vessels and the achievement of the target flow range.

## Conclusion

This study developed and demonstrated the successful use of an *ex vivo* test setup in which human umbilical cords can be effectively and reproducibly cannulated and operated at physiological flow conditions. The *ex vivo* test setup closes the gap between existing animal models and the human neonate and thereby offers a low-risk and ethical method to investigate cannulation devices and procedures for AAPT applications and umbilical cord-related therapies in general.

## Data Availability

The raw data supporting the conclusions of this article will be made available by the authors, without undue reservation.
