# Supplementary material for: Reperfused human umbilical cords as an ex vivo model for ECMO cannulation in artificial placenta technology
Source: Front Bioeng Biotechnol. 2025 Oct 21;13:1663442. doi: 10.3389/fbioe.2025.1663442 (PMC12583208; doi:10.3389/fbioe.2025.1663442)
Supplement: Supplementary file 1 [file Table1.docx]

| No. | Cannula size | Perfusion time of umbilical cord in min |
| --- | --- | --- |
| 1 | 1xA+1xV 5Fr (A) 7Fr (V) Radiofocus Introducer II Pediatric Kit B Terumo | 40 |
| 2 | 1xA+1xV 6Fr (A) 7Fr (V) Radiofocus Introducer II Pediatric Kit B Terumo | 45 |
| 3 | 1xA+1xV 6Fr Radiofocus Introducer II Pediatric Kit B Terumo | 50 |
| 4 | 1xA+1xV 6Fr (A) 7Fr (V) Radiofocus Introducer II Pediatric Kit B Terumo | 30 |
| 5 | 1xA+1xV 6Fr (A) 7Fr (V) Radiofocus Introducer II Pediatric Kit B Terumo | 55 |
| 6 | 2xA+1xV 5Fr+6Fr Radiofocus Introducer II Pediatric Kit B Terumo | 50 |
| 7 | 2xA+1xV 6Fr+7Fr Radiofocus Introducer II Pediatric Kit B Terumo | 45 |
| 8 | 2xA+1xV 6Fr+7Fr Radiofocus Introducer II Pediatric Kit B Terumo | 45 |
| 9 | 2xA+1xV 6Fr+7Fr Radiofocus Introducer II Pediatric Kit B Terumo | 95 |
| 10 | 2xA+1xV 6Fr+7Fr Radiofocus Introducer II Pediatric Kit B Terumo | 55 |
| 11 | 2xA+1xV 6Fr+7Fr Radiofocus Introducer II Pediatric Kit B Terumo | 55 |
| 12 | 2xA+1xV 6Fr+7Fr Radiofocus Introducer II Pediatric Kit B Terumo | 45 |
| 13 | 2xA+1xV 6Fr+7Fr Radiofocus Introducer II Pediatric Kit B Terumo | 45 |
| 14 | 2xA+1xV 6Fr+7Fr Radiofocus Introducer II Pediatric Kit B Terumo | 50 |
| 15 | 2xA+1xV 6Fr+7Fr Radiofocus Introducer II Pediatric Kit B Terumo | 45 |
